# Recent progress on smart lower prosthetic limbs: a comprehensive review on using EEG and fNIRS devices in rehabilitation

**DOI:** 10.3389/fbioe.2024.1454262

**Published:** 2024-08-26

**Authors:** Nouf Jubran AlQahtani, Ibraheem Al-Naib, Murad Althobaiti

**Affiliations:** ^1^ Biomedical Engineering Department, College of Engineering, Imam Abdulrahman Bin Faisal University, Dammam, Saudi Arabia; ^2^ Bioengineering Department, King Fahd University of Petroleum & Minerals, Dhahran, Saudi Arabia; ^3^ Interdisciplinary Research Center for Communication Systems and Sensing, King Fahd University of Petroleum & Minerals, Dhahran, Saudi Arabia

**Keywords:** neurorehabilitation, electroencephalography (EEG), brain-computer interfaces (BCIs), functional near-infrared spectroscopy (fNIRS), lower prosthetic limbs

## Abstract

The global rise in lower limb amputation cases necessitates advancements in prosthetic limb technology to enhance the quality of life for affected patients. This review paper explores recent advancements in the integration of EEG and fNIRS modalities for smart lower prosthetic limbs for rehabilitation applications. The paper synthesizes current research progress, focusing on the synergy between brain-computer interfaces and neuroimaging technologies to enhance the functionality and user experience of lower limb prosthetics. The review discusses the potential of EEG and fNIRS in decoding neural signals, enabling more intuitive and responsive control of prosthetic devices. Additionally, the paper highlights the challenges, innovations, and prospects associated with the incorporation of these neurotechnologies in the field of rehabilitation. The insights provided in this review contribute to a deeper understanding of the evolving landscape of smart lower prosthetic limbs and pave the way for more effective and user-friendly solutions in the realm of neurorehabilitation.

## 1 Introduction

Lower limb amputations are relatively common, particularly among patients with diabetes, peripheral artery disease, severe trauma, and infections. Each year, around 1 million lower limb amputations are performed worldwide, with a significant portion of these occurring in the diabetic population (Ziegler-Graham et al., 2008; Marcinkowska et al., 2021). In the United States alone, about 185,000 amputations occur annually, the majority of which are lower limb amputations (Asif et al., 2021; Jeffcoate et al., 2021). The impact on patients’ quality of life is profound and multifaceted. Physically, patients face challenges with mobility and functionality, often experiencing pain and phantom limb sensations. Mentally, the psychological impact can be severe, leading to depression, anxiety, and post-traumatic stress disorder. Socially, amputees often encounter stigma and barriers to participation in social and recreational activities (Moxey et al., 2011).

The economic impact is substantial, encompassing high medical costs. For diabetic patients, lifetime costs can exceed $500,000, including the initial surgery, rehabilitation, and prosthetic devices. Additionally, many amputees struggle to return to work, resulting in reduced income and financial instability (Alessa et al., 2022; Ezzatvar and García-Hermoso, 2023). Therefore, there is a critical need for advancements in prosthetic technology and ensuring these advancements are accessible to all amputees. Providing patients with the best possible prosthetic devices can significantly enhance their quality of life.

To enhance the quality of life for amputees, recent years have seen significant innovations in the domain of smart lower prosthetic limbs (Safari, 2020; Asif et al., 2021; Pană et al., 2022). Current prosthetic limbs have come a long way, but there are still limitations. Traditional prosthetics rely on surface electromyography (EMG) to detect muscle activity for control, which can be limited by residual limb movement and fatigue. Notably, technologies such as electroencephalography (EEG) and functional near-infrared spectroscopy (fNIRS)—traditionally utilized as neuroimaging as well as brain-computer interfaces (BCIs) techniques—are now being implemented in prosthetics (Murphy et al., 2017; Khan R. A. et al., 2018). The integration of EEG and fNIRS into prosthetic limb designs is forging paths toward restoring not just lost functionality, but also intuitive control and a deeper connection between amputees and their artificial limbs, promising a future where enhanced autonomy is a reality (Kwon et al., 2020; Bourguignon et al., 2022; Chen et al., 2023). This review paper explores the integration of EEG and fNIRS technologies for advancing lower limb prosthetic control. Key aspects covered include the potential benefits of a hybrid BCI, recent advancements, and promising results. The paper concludes by offering a thorough comparison summarizing the advantages and disadvantages of recently selected papers that used hybrid systems as well as recommendations for future research and discuss broader implications for rehabilitation and assistive technology.

Today, prostheses have evolved, incorporating cutting-edge technology to enable effective limb control. This integration, achieved by merging computer-based technologies with electronic systems, allows for sophisticated levels of prosthesis management. When a prosthesis is equipped with an electronic system, it is termed a bionic prosthesis. Such devices employ various techniques to ensure comprehensive control of the prosthetic limbs through electronic technology. This review concentrates on two specific systems: EEG and fNIRS, both instrumental in controlling prostheses. EEG monitors electrical activity within the brain, while fNIRS measures the hemodynamic changes in the brain, i.e., oxygenation and blood flow (Khan R. et al., 2018; Hosni et al., 2020). They are useful for brain-computer interfaces and in the management of prosthetic limbs.

This paper delves into the recent advancements in smart lower prosthetic limbs, emphasizing the critical role played by EEG and fNIRS technologies. Prosthetics equipped with EEG have the potential to interpret the electrical signals from the user’s brain, facilitating natural and fluid limb movements, and markedly improving the quality of life for amputees. Additionally, fNIRS can provide vital insights into the brain’s hemodynamic responses. Through a comprehensive review of the existing literature, we aim to provide an overview of the current state of the art, challenges, and prospects in the field of smart prosthetics driven by EEG and fNIRS. Moreover, we delve into the historical context and evolution of prosthetic limbs, highlighting the limitations of traditional designs. We discuss the fundamental principles of EEG and fNIRS technologies and their relevance in bridging the gap between the human brain and artificial limbs. Furthermore, we address the key challenges in the implementation of EEG-driven prosthetics, such as signal processing, robustness, and user training. Finally, we explore the potential applications, including enhanced mobility, proprioception, and the creation of a direct neural interface with the artificial limb. Table 1 illustrates the advantages and challenges of EEG and fNIRS in this context.

**TABLE 1:** Advantages and challenges of EEG and fNIRS in controlling prosthetic limbs (Jungnickel et al., 2019; Gao et al., 2020; Crum, 2021; Liu et al., 2021; Ortega and Faisal, 2021; Uchitel et al., 2021; Padfield et al., 2022; Kotwal et al., 2023; Vyas et al., 2023).

Criterion	Sub-criterion	EEG	fNIRS
Signal Quality	Brain wave detection	• EEG technology captures relevant brain wave signals of an intention ^a^ • EEG electrodes can measure electrical brain activity	• fNIRS technology monitors variations in hemoglobin concentration (oxyhemoglobin and deoxyhemoglobin) in the brain. Consequently, hemoglobin concentration changes can be used to analyze neural activity in the cortex • fNIRS optodes can measure brain hemodynamics
	Resolution	• EEG offers quite high temporal resolution	• fNIRS offers better spatial resolution compared to EEG signals, leading to precise localization of brain activity and finer-grained control over prosthetic movements
	signal clarity	• EEG signals feature low amplitudes, typically measured in microvolts	• fNIRS offers good signal-to-noise ratio (SNR)
Usability	Intuitive control ^b^	• EEG-equipped prosthetics interpret and translate brain waves signals into action • Users can control their prosthetic limbs through their thoughts	• fNIRS-equipped prosthetics interpret and translate hemoglobin concentration changes signals into action • Users can control their prosthetic limbs through their thoughts
	Learning curve	•Users become experts in generating brain signals related to desired movements after training sessions • EEG-equipped prosthetic systems can adapt to their brain activity	• fNIRS needs a less extensive training period compared to EEG. • fNIRS can be used to control prosthetic limbs without susceptibility to electrical noise
System Efficiency	Accuracy	• EEG signals are highly susceptible to noise, such as muscle noise and power line interferences	• fNIRS can be integrated with different technologies using multimodal BCI approaches • Some researchers combine EEG and fNIRS to boost the robustness and accuracy of control systems
	Brain-computer-interface capabilities	• EEG integration with BCI establishes a link between the user’s brain and the prosthetic limb	• fNIRS integration with BCIs establishes a link between the user’s brain and the prosthetic limb
	Latency	• EEG signals are direct measures of brain activities, i.e., faster detection of brain activities	• fNIRS detects changes in hemoglobin concentration, which are indirect measures of brain activities, i.e., slower detection of brain activities
Functional Capability	Task performance	• Users can sense their prosthetic limb position and movement in real-time	• Users can adjust and refine their control over the prosthetic limb through training and practice
	Real-time adaptability	• Some EEG-driven prosthetics include sensors and feedback mechanisms to boost control and coordination of prosthetic limbs	• fNIRS cannot be used in real-time applications
	Real-world Applications	• EEG-driven prosthetic limbs can be used in various fields, such as rehabilitation • EEG-driven prosthetic limbs can be integrated into gaming systems and used in virtual reality	• fNIRS can be used to control prosthetic limbs • fNIRS can be integrated into wearable robotics, exoskeletons, and assistive devices for enhanced mobility and functionality

^a^ This term refers to specific patterns in brain activity that are indicative of the user’s intention to perform a particular action or task. These signals are typically identified using neuroimaging techniques and are crucial for understanding and interpreting the user’s mental state or commands in brain-computer interface systems.

^b^ Intuitive control refers to a control system designed to be naturally and easily operated by users with minimal learning or effort. In the context of BCIs, it means that the system interprets the user’s brain signals in such a way that the user can control it without requiring extensive training or conscious thought about the interaction process.

## 2 Anatomy and physiology of the lower limb movements during the gait cycle

**FIGURE 1:**
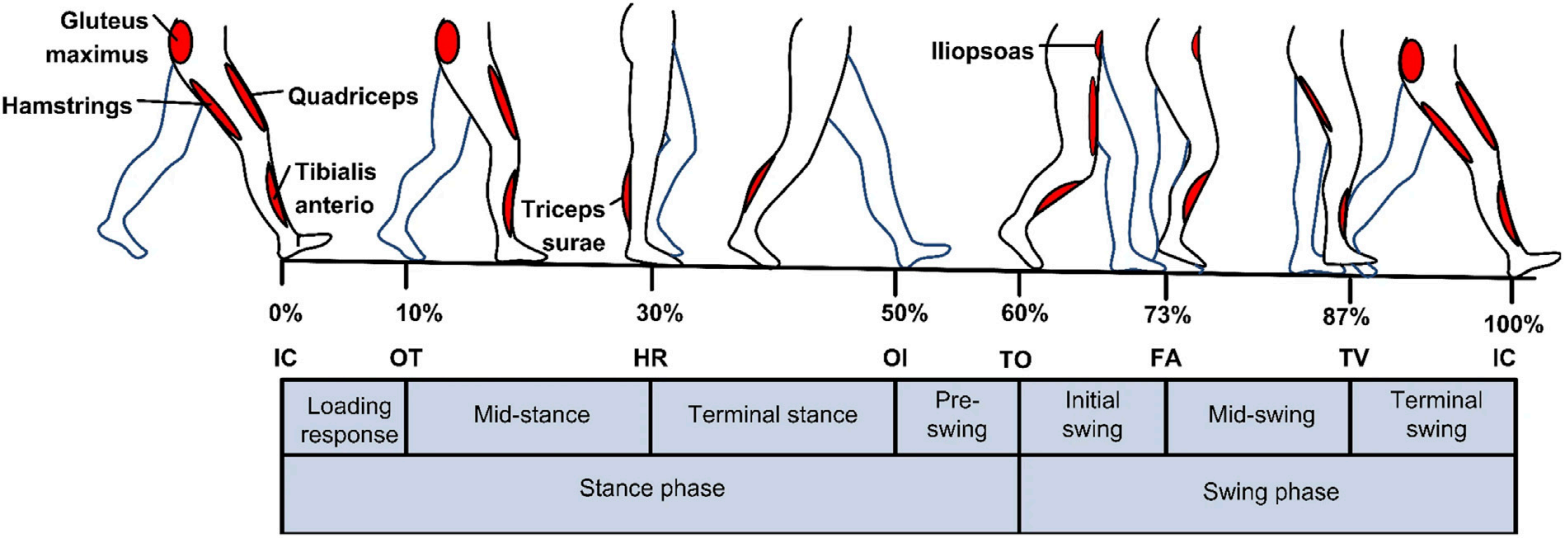
An illustration of how lower limb muscles are controlled by central nervous system during the gait cycle. Reprinted with permission from Ref. (Al-Shuka et al., 2019). The figure illustrates the two walking phases: The stance and Swing phases. Sub-phases of the Stance phase are indicated in the figure: initial contact IC, opposite toe off OT, heel rise HR, and opposite initial contact OI. Sub-phases of the Swing phase are toe-off TO, feet adjacent FA, and tibia vertical TV.

Physiological signals that initiate movements in the lower limb are coordinated by a complex interplay between the nervous system and the musculoskeletal system. The central nervous system, primarily the brain and the spinal cord, plays a vital role in transmitting signals to the lower limb muscles to initiate and control movement during the gait cycle (Ting et al., 2015). Figure 1 shows the gait cycle with corresponding lower limb muscles. When an individual decides to take a step, the brain sends signals through the motor cortex, which is responsible for planning and executing voluntary movements. These signals travel down the spinal cord within a bundle of nerve fibers called the corticospinal tract. The corticospinal tract carries the motor commands from the brain to the lower motor neurons located in the ventral horn of the spinal cord (Ting et al., 2015).

Upon reaching the lower motor neurons, these signals are distributed to specific muscles involved in walking. For example, the muscles responsible for extending the leg during the stance phase and flexing the leg during the swing phase are activated by these signals. This activation occurs through a process called neuromuscular transmission, where the lower motor neurons release chemicals called neurotransmitters that stimulate the muscles to contract and generate movement (Ting et al., 2015; Al-Shuka et al., 2019). In addition to the motor commands sent by the brain, the nervous system also receives feedback from sensory receptors located in the lower limb. These receptors, known as proprioceptors, relay information about joint angles, muscle length, and tension to the brain. This feedback loops back to the brain, allowing it to monitor and adjust the movements during the gait cycle (Ting et al., 2015; Al-Shuka et al., 2019). Furthermore, the brain continuously monitors and integrates information from various sensory systems, such as the visual and vestibular systems, to maintain balance and coordination during walking. This integration occurs in specialized areas of the brain, including the cerebellum, basal ganglia, and brainstem, which play crucial roles in modulating and refining the motor commands sent to the lower limbs (Ting et al., 2015; Al-Shuka et al., 2019). Overall, the brain’s control over the gait cycle is a complex process involving the initiation of movement signals, the integration of sensory information, and the continuous adjustment and coordination of lower limb muscles. By understanding the intricate interplay between the brain and the lower limb, researchers can gain insights into disorders affecting gait and develop interventions to optimize motor control and promote efficient walking patterns (Ting et al., 2015; Al-Shuka et al., 2019; Lacerenza et al., 2021; Yokoyama et al., 2021).

Electroencephalography and functional near-infrared spectroscopy are two neuroimaging techniques that offer a direct link to the brain’s electrical and hemodynamic activities, respectively (Chen et al., 2020; Liu et al., 2021; Yokoyama et al., 2021). EEG measures the electrical activity generated by the firing of neurons in the brain, providing high-temporal resolution insights into the neural dynamics underlying cognitive and motor processes. On the other hand, fNIRS detects changes in blood oxygenation within the cortex, offering a spatially informed view of cerebral blood flow and metabolic activity. The complementarity of these methods lies in their combined ability to capture both the timing (from EEG) and the localized blood-based neural responses (from fNIRS). This simultaneous recording can be especially beneficial in real-world applications like brain-computer interfaces or in clinical settings for neurological rehabilitation, where understanding both the immediate neural reactions and the longer-lasting metabolic changes is crucial.

## 3 Materials and methods

**FIGURE 2:**
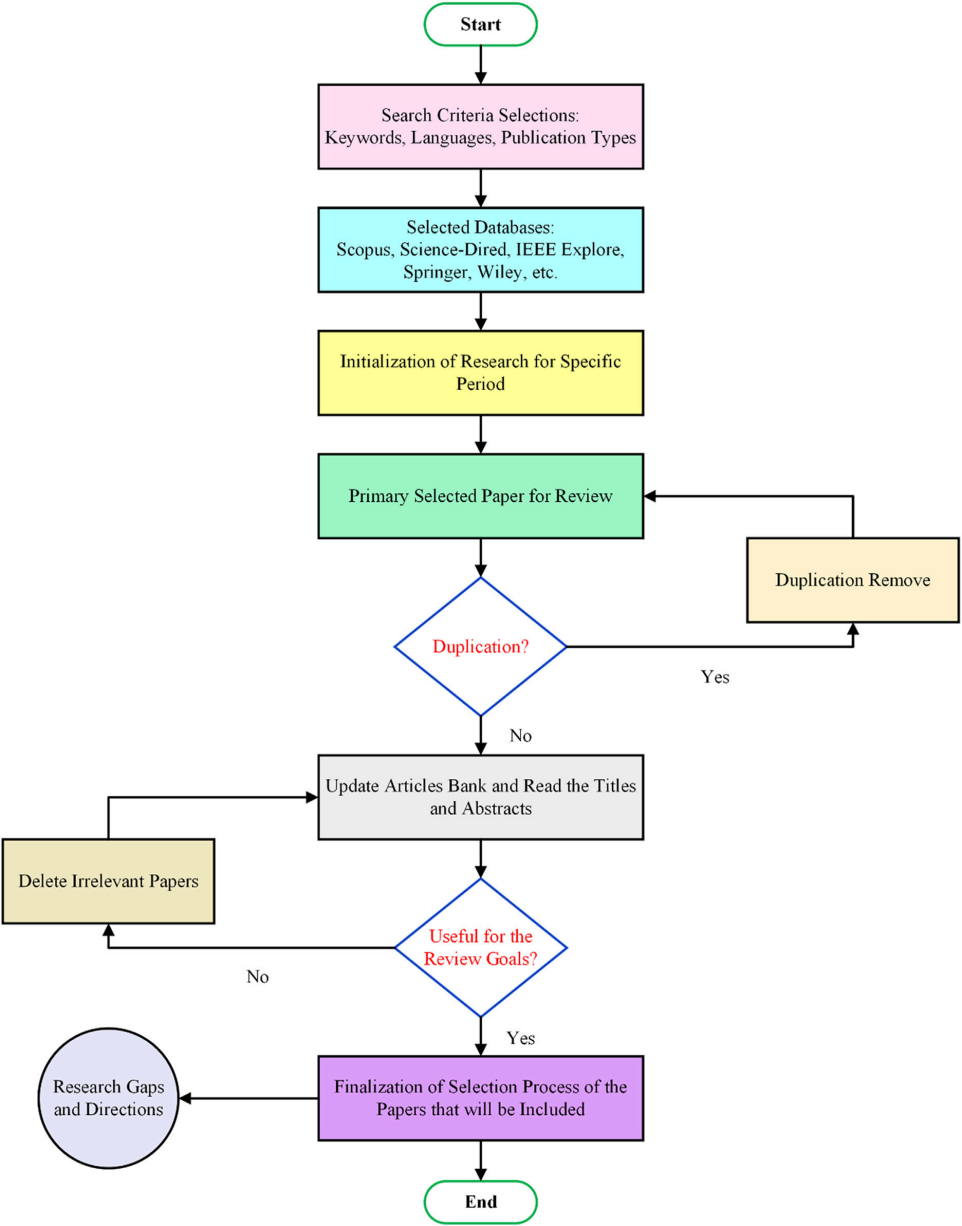
Flowchart of the selection process of papers used in this review.

To conduct any review about a specific topic, it is critical to specify a specific approach to determine relevant papers. The adopted methodology in this review is shown in Figure 2. Firstly, the search selection criteria, such as research keywords: electroencephalography (EEG), brain-computer interfaces, functional near-infrared spectroscopy (fNIRS), smart prostheses, gait rehabilitation), languages, and publication type, were defined for the period from 2016 till now. Studies were included if they met the following criteria: (1) the study was relevant to the review topic based on the assessment of the abstract and the keywords; (2) the study was accessible in the English language or could be easily translated to another language using available translators; (3) the study published as a review paper or article. Next, well-known and trustworthy publishers such as Science Direct, IEEE, Springer, and Wiley were chosen to find the relevant papers. Similarly, the keywords were used to limit the search boundaries throughout the search stage. Since the research quality is a crucial parameter when conducting the research; papers published in ISI and prestigious journals were considered to guarantee the research quality. The initial search using the review methodology yielded many papers. Only the relevant and unduplicated papers were sorted based on their titles, abstracts, and contributions. The final list of papers was then screened and reviewed accordingly.

The keywords used to search the literature are: fNIRS, EEG, prosthetics, lower limb, BCI, gait rehabilitation, and hybrid fNIRS-EEG. Using these keywords, there were 137 found during the search. Among that, there were 105 papers shortlisted that satisfied the criteria. Following the removal of duplicates, 98 papers were included in the final review.

## 4 Prosthetic limb EEG-Based control

EEG is a versatile, non-invasive tool essential in diagnosing and managing neurological and psychiatric conditions, aiding in clinical and surgical procedures, neonatal care, and cognitive research (Shoka et al., 2019). The EEG-based control system for prosthetic limbs derives its functionality from the brainwaves captured by EEG devices, which are then translated into executable commands through a brain-computer interface. The central concept of BCI technology is to establish a communication channel that translates brain activity patterns into mechanical actions for prostheses. EEG acquisition, as a technique, is known for its cost-effectiveness and efficiency. It involves positioning multiple electrodes on the patient’s scalp to detect cerebral signals (Al-Quraishi et al., 2018). However, one of the challenges in using EEG is its susceptibility to electric noise, including bioelectrical signals such as the ECG, resulting in a relatively low signal-to-noise ratio (SNR). Consequently, the EEG signals are characterized by their low amplitudes, typically measured in microvolts, and their frequency range, which spans from 1 Hz to 100 Hz. To capture EEG signals, non-invasive methods are mostly used and invasive methods using specialized electrodes that are attached to the cortex or even inserted inside the brain can be used as well in rare cases (Al-Quraishi et al., 2018). Nevertheless, our review focuses exclusively on non-invasive techniques, which are more commonly adopted due to their safety and ease of use. Invasive neuroimaging offers precise control signals for prosthetics but includes risks like infection and complex surgery. Non-invasive methods, such as EEG and fNIRS, are safer and easier to manage but offer lower signal resolution. The choice between them balances signal quality against safety and practicality.

### 4.1 EEG-based control architecture

Numerous methods are employed to orchestrate the movement of prosthetic limbs, yet the non-invasive EEG approach stands out for its direct and substantial control capabilities. This system harnesses brain signals to guide the movement of the prosthetic, transforming cerebral activity into actionable commands via BCI technology. Consequently, amputees gain a sense of regained autonomy, akin to their natural limb functionality. Moreover, this technology allows them to perceive touch sensations with the prosthetic limb (Murphy et al., 2017).

**FIGURE 3:**
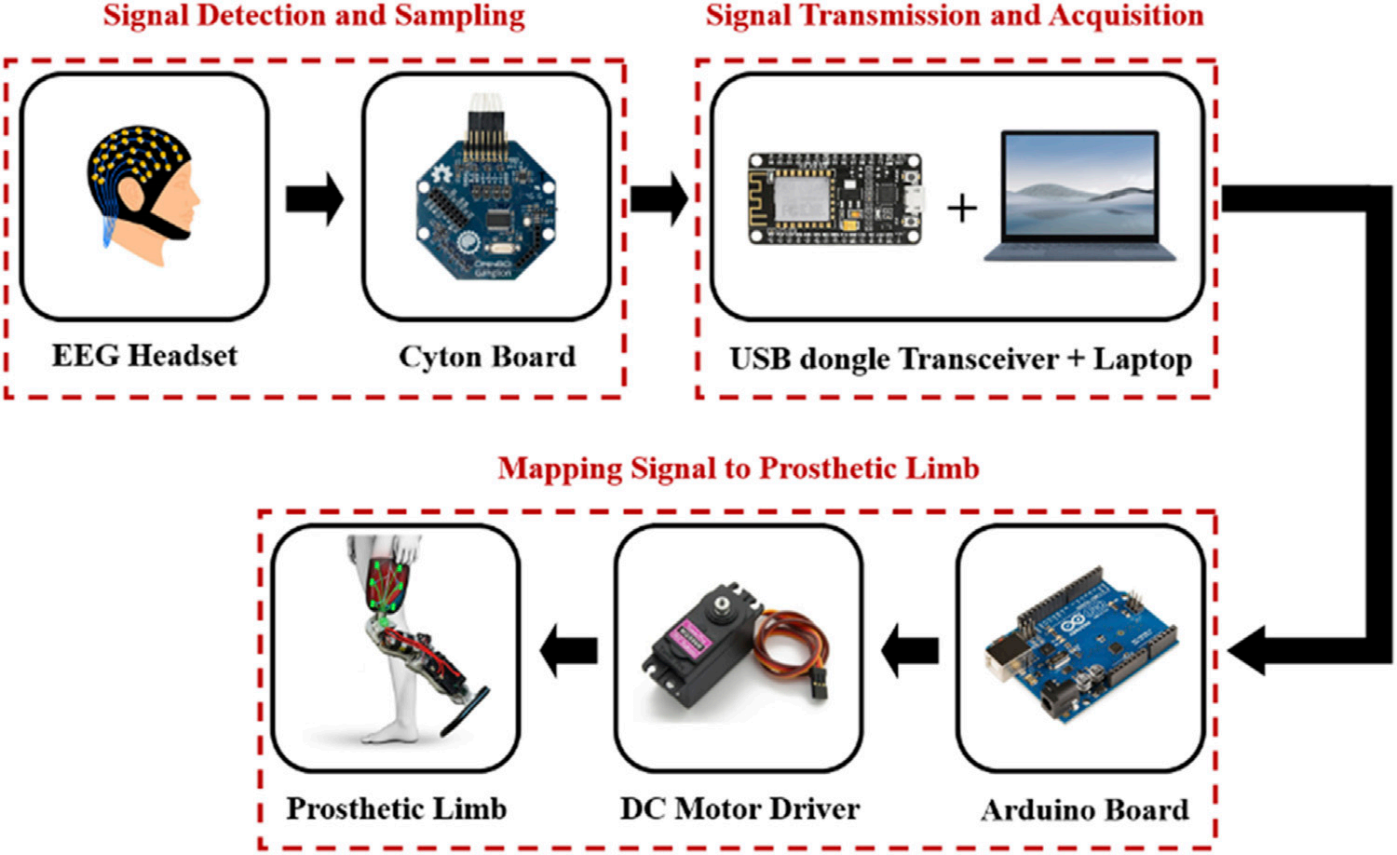
Block diagram of an EEG-based control for a prosthetic leg.

Figure 3 illustrates the architecture of an EEG-based, brain-controlled prosthetic leg, delineating the core stages inherent to various EEG-based control system designs. These stages comprise (A) Signal Detection and Sampling (B) Signal Transmission and Acquisition, and (C) Mapping of the Signal to the Prosthetic Leg. The specifics of each stage will be elaborated in the subsequent sections (Murphy et al., 2017; Al-Quraishi et al., 2018).

#### 4.1.1 Signal Detection and Sampling

Current EEG electrode technology, especially the conventional Ag/AgCl wet electrodes, has its fair share of limitations despite providing a high signal-to-noise ratio (SNR). While Ag/AgCl electrodes are proficient in detecting EEG signals with high accuracy, they necessitate conductive gel and meticulous skin preparation, making the process lengthy and unsuitable for prolonged signal acquisition (Al-Quraishi et al., 2018; Bansal and Mahajan, 2019; Soufineyestani et al., 2020; Gu et al., 2021; Hu et al., 2021; Yuan et al., 2021). To solve these issues, various dry electrodes have been explored. For instance, Liu et al. (2019) demonstrated significant progress with the introduction of nano-modified dry electrodes. These novel electrodes came in two lengths to cater to various head regions, striking a balance between user comfort and signal quality. After extensive comparison and clinical testing, Li et al. (2021) concluded that these electrodes could feasibly replace traditional Ag/AgCl wet electrodes. However, they emphasized the need for further evaluation across varied mental tasks and demographics. In a notable achievement, Li et al. (2021) developed a polyacrylamide/polyvinyl alcohol super-porous hydrogel-based semi-dry electrode, particularly effective for EEG recording on hairy scalps. This new electrode showcased significant potential, marked by a temporal cross-correlation coefficient of 0.941 compared to conventional wet electrodes. Recent advancements by Fiedler et al. (2015) introduce dry electrodes made of polyurethane coated with Ag/AgCl, significantly enhancing the reliability of EEG signal detection. Other technologies, such as MEMS and 3D printing, facilitate the development of dry electrodes but come with their challenges, such as structural fragility and rigidity, respectively.

Table 2 compares wet and dry EEG electrodes and highlights advancements in dry electrode technology. While traditional wet electrodes use conductive gels for improved signal quality and lower impedance, dry electrodes negate the need for such gels, enhancing convenience and portability. However, dry electrodes tend to have higher impedance and susceptibility to movement artifacts, although at lower impedances, they generate less noise than wet electrodes. Another important aspect that plays a significant role in choosing the electrodes is whether to choose active or passive electrodes. They differ based on signal handling; active electrodes pre-amplify signals at the scalp for reduced transmission noise, whereas passive electrodes amplify signals at the endpoint, highlighting trade-offs in cost and noise potential. Choosing the appropriate electrode type—wet/dry and passive/active—necessitates balancing signal quality and convenience, tailored to specific experimental or clinical needs. In summary, while traditional wet electrodes like Ag/AgCl are effective, newer innovations in dry and semi-dry electrodes are paving the way for more efficient, comfortable, and longer-term EEG signal acquisition (Lopez-Gordo et al., 2014; Fiedler et al., 2015; Hinrichs et al., 2020).

**TABLE 2:** Comparison between dry and wet electrodes at higher and lower impedances (Hinrichs et al., 2020).

	At low impedances		At high impedances	
	Dry electrodes	Wet electrodes	Dry electrodes	Wet electrodes
Mean	10.0 µV2	33.1 µV2	45.0 µV2	11.1 µV2
Median	2.6 µV2	3.9 µV2	4.9 µV2	3.2 µV2
SD	30.5 µV2	121.4 µV2	189.0 µV2	21.4 µV2
Z	−4.98	−4.98	3.36	3.36
P	0.0001	0.0001	0.0008	0.0008

**FIGURE 4:**
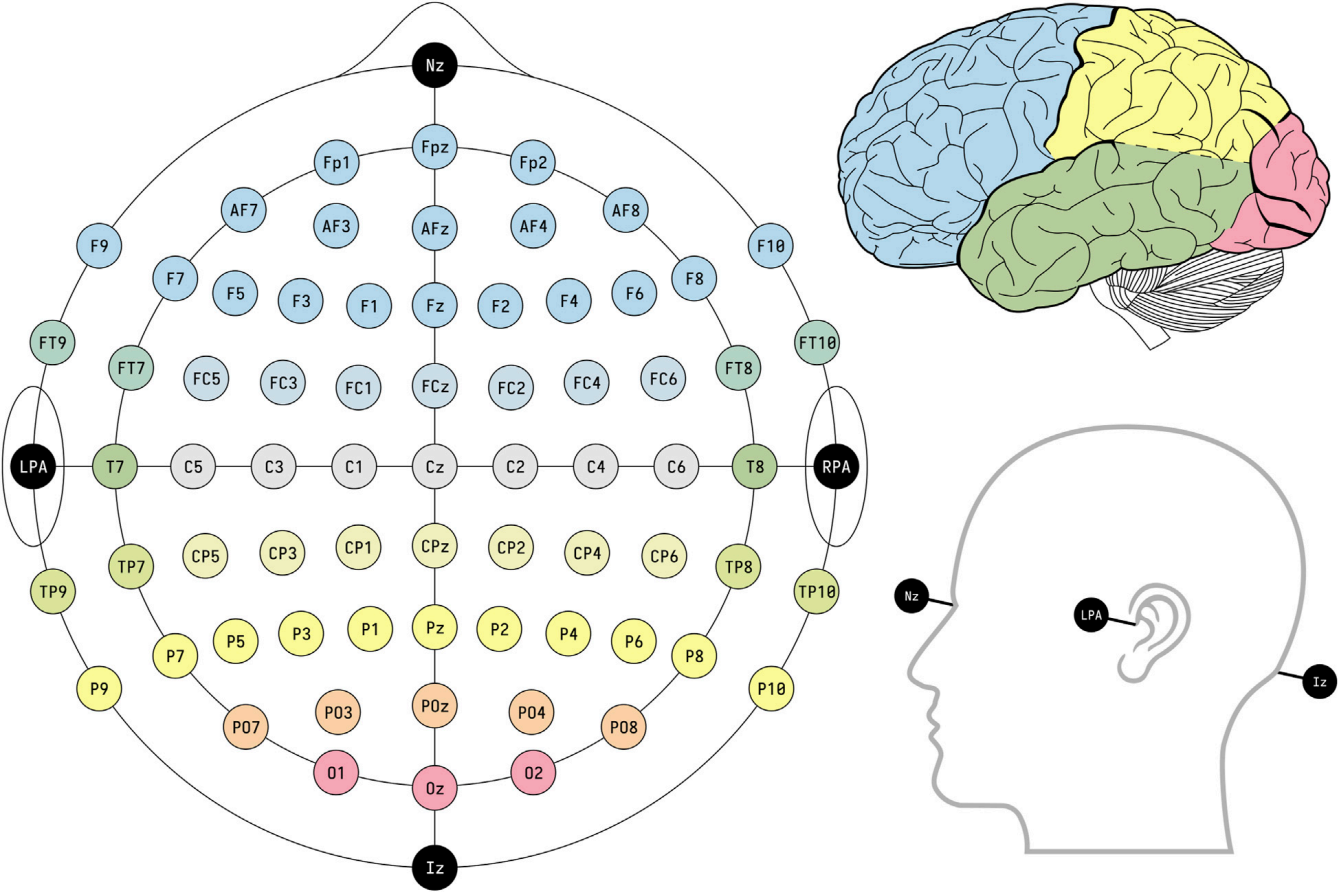
EEG electrode placement based on the international standard framework 10–20. Reprinted with permission from (BruceBlaus, 2024).

To accurately capture the desired signals, it’s imperative that the electrodes be positioned over the area corresponding to the signal’s origin. For prosthetic limb control applications, electrodes are strategically situated on the motor cortex, the part of the brain responsible for generating movement-related signals (Casson et al., 2018; Arpaia et al., 2023). Highlighting the significance of electrode placement, a study by Domingos et al. (2018) determined that selecting electrode locations tailored to the specific application boosts the reliability of the results, thereby enhancing EEG system efficacy. Typically, the arrangement of electrodes adheres to the 10–20 international system, the layout of which is depicted in Figure 4.

The minute electrical signals produced by the human brain necessitate amplification to discern voltage variations for analytical interpretation. Amplifiers fulfil this role by intensifying the input voltage, in the order of tens of microvolts, multiplying it by factors that can reach up to one million. Recent studies have explored the development of innovative amplifiers tailored to EEG systems (Karacaoğlan and Tokmakçi, 2017; Portelli and Nasuto, 2017; Lee et al., 2019a; Lin et al., 2019; Shad et al., 2020).

#### 4.1.2 Signal Transmission and Acquisition

**FIGURE 5:**
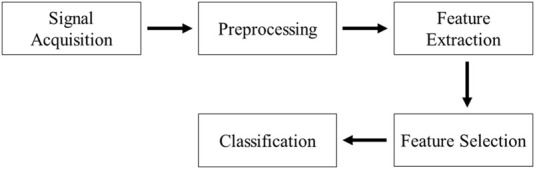
The block diagram of used method to use brainwave for prosthesis control.

Digitized EEG signals are transmitted wirelessly to a computer via the OpenBCI USB dongle, which serves as a transceiver. The raw brainwave acquisition process is executed using OpenBCI software. This allows for the visualization of raw EEG data during the recording phase and facilitates its storage on the computer in a text format. Subsequently, this stored data undergoes pre-processing, wherein it is cleaned and filtered, and is then utilized for the classification process through specialized software such as MATLAB or Python. Figure 5 illustrates the general brainwave processing approach employed for prosthetic control (Hassan and Hussain, 2023).

Through specialized computer software, raw EEG data is filtered to eliminate noise and artifacts that affect the integrity of the signal. These artifacts may originate from both internal and external sources, potentially misrepresenting the true brain signal. Any recorded event not originating from the brain is considered noise or an artifact. These can be divided into two categories: physiological and extra-physiological artifacts. Physiological artifacts, generated internally, include those from electromyography (EMG), electrooculography (EOG), and electrocardiography (ECG). In contrast, extra-physiological artifacts arise from external environmental factors. There are various techniques for noise removal, with Wiener and adaptive filtering being among the primary methods. In adaptive filtering, the extent of contamination is initially assessed by iteratively adjusting weights through an optimization algorithm. The resultant noise estimate is then subtracted from the original EEG signals. On the other hand, Wiener filtering is a linear statistical approach aimed at closely approximating the real EEG signal. It operates on the principle of linear time-invariance to minimize the mean square error between the true and estimated signals. This method’s linearity is achieved by calculating the power spectral density of both the measured and artifact signals. However, the implementation of Wiener filtering presents challenges, particularly the requirement for periodic calibration as indicated in the references (Elsayed and Bayoumi, 2017; Shoka et al., 2019).

After EEG signals are processed, features are extracted from the filtered data for signal classification. Distinctive features are chosen for their efficacy in accurately describing varying EEG signals, guided by statistical measures defined by the programmer. Commonly used features include time-domain measures and frequency band power features. Before training a model, it is essential to assess the significance of the available data volume. A limited dataset can lead to overfitting, resulting in data misrepresentation and incorrect outcomes. Following feature extraction, these features are employed by chosen classifiers—such as artificial neural networks, logistic regression, support vector machines, and linear discriminant analysis—to interpret and execute commands for application use. The classifier’s overriding objective is to differentiate between distinct classes, as referenced in (Arpaia et al., 2023).

#### 4.1.3 Mapping signal to prosthetic leg

To control a prosthetic leg using brainwaves, signals are relayed from the computer to a microcontroller, such as the Arduino Uno, through Bluetooth (Murphy et al., 2017). The Arduino board is a cost-effective, open-source microcontroller that is programmable via the Arduino Software (IDE). It operates through a USB cable connected to a laptop or an external battery. The Arduino Uno interprets brainwave data to produce appropriate movement commands. These commands are used by the DC motor within the prosthetic limb to modulate the prosthetic leg’s speed, position, and direction. Additionally, the biocompatibility of prosthetic materials is critical to prevent adverse biological reactions, such as toxicity (Brack and Amalu, 2021).

### 4.2 EEG technology: new controlling approaches

**FIGURE 6:**
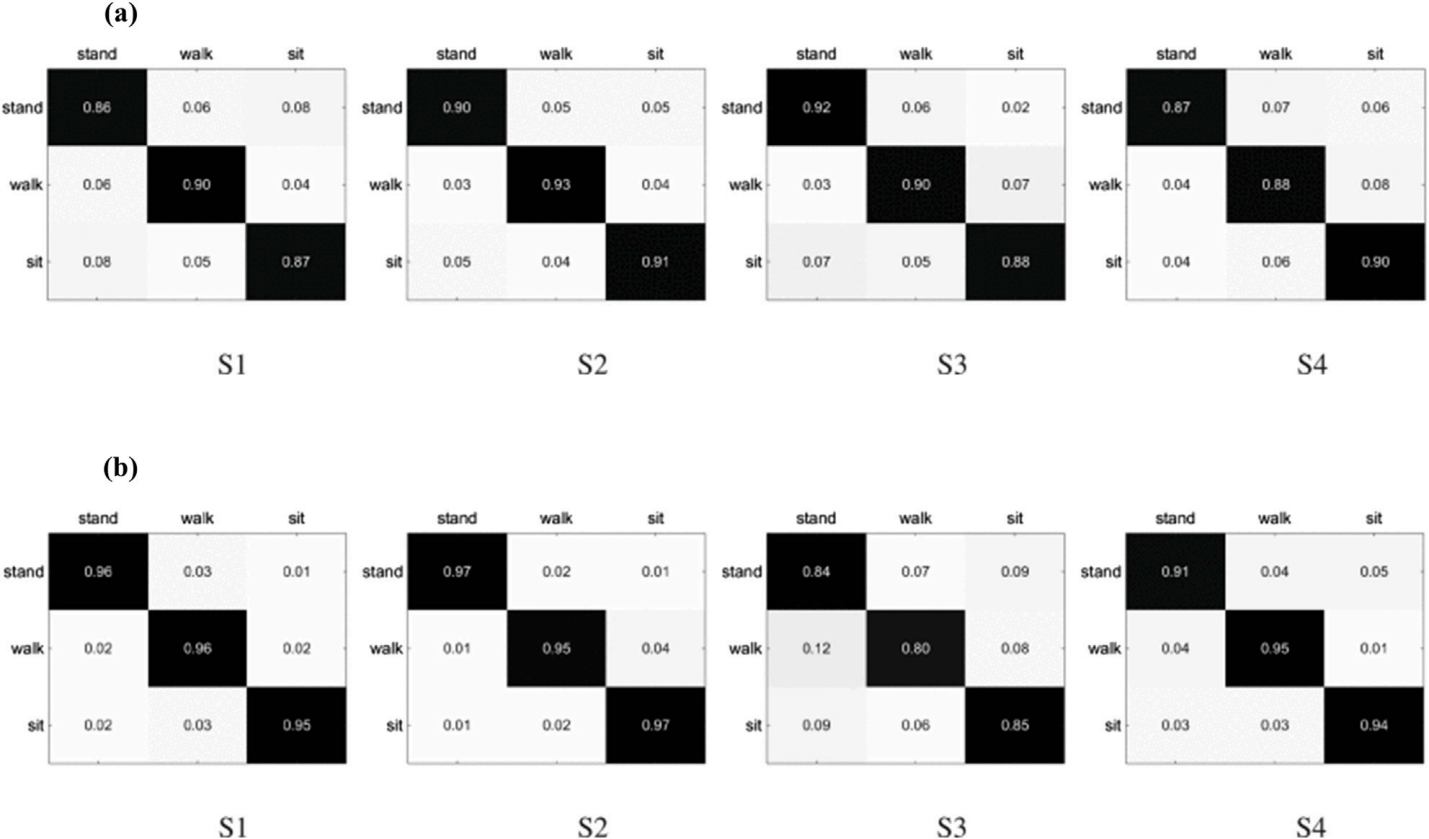
Classification results of four basic movements obtained from subjects (S1, S2, S3, and S4) using **(A)** SSVEP and **(B)** motor imagery BCIs. Reprinted with permission from (Wang et al., 2018).

Recent research aims to predict an individual’s lower limb movements via EEG signals to enhance control over prosthetic limbs. Wang et al. (2018) documented a novel multimodal method to manipulate a lower-limb exoskeleton, with four subjects participating in the study. This exoskeleton adjusted to the user’s motion intentions decoded from EEG signals. Subjects executed basic motions such as walking forward, standing up, and sitting down. The study also tested two different Brain-Computer Interface systems: one relying on steady-state visual evoked potentials (SSVEP) and the other on motor imagery. As depicted in Figure 6, the results demonstrated high accuracy rates, achieving 90% for SSVEP and between 94% and 97% for motor imagery, in classifying the intended motion tasks (Wang et al., 2018). However, Subject 3 (S3) yielded lower classification accuracies with motor imagery BCI; this disparity may reflect the inherent variability of EEG signals across individuals and their susceptibility to numerous influencing factors.

Hasan (2022) explored the potential of pre-emptively identifying gait intention, a vital element in crafting and actualizing prosthetics. Prompt recognition of gait intention enables the timely calibration of prosthetic system settings to align with user demands. The study spanned diverse terrains: asphalt, brick, concrete, grass, and gravel. The researchers utilized an ankle-mounted camera, an onboard chip, and a low-power Raspberry Pi unit to achieve their objectives. Their findings revealed exceedingly precise forecasting of terrain types and transitions, demonstrating the capacity to anticipate an imminent terrain change of 0.5 µs–1.3 µs prior to taking the first step onto new terrain. This research markedly advances the development of lower prostheses that mimic natural limb function (Hasan, 2022).

Zhang et al. (2022) presented a machine-learning algorithm adept at discerning human motion intentions through sensor data, such as displacement, force, and velocity of wheel movement. This innovation holds significant promise for enhancing the synergy between humans and robotic assistance. Employing a bi-directional long short-term memory model, the system accurately identifies various actions—walking, turning, falling—with a stellar accuracy rate of 99.61%. In a parallel approach, they utilized a radial basis function neural network coupled with an adaptive sliding mode controller (RBFNN-ASMC) to capture and adjust for a patient’s behavioral intentions. The RBFNN-ASMC controller demonstrated superior gait correspondence compared to traditional PID controllers, as depicted in Table 3 (Zhang et al., 2022).

**TABLE 3:** Accuracy and elapsed time of the three algorithms for human activity events. Reprinted with permission from (Zhang et al., 2022).

	Maximum error		Average error		Standard deviation	
	Hip	Knee	Hip	Knee	Hip	Knee
PID	16.718°	4.556°	1.405°	1.822°	2.235°	1.497°
RBFNNASMC	16.628°	2.996°	0.197°	0.037°	1.486°	0.269°

### 4.3 Advantages and disadvantages of EEG for controlling prosthetic

Although EEG signals are integral in prosthetic limb control due to their high temporal resolution, safe, non-invasive acquisition, and the variety of features they offer, they come with a trade-off in spatial resolution and are prone to interference from artifacts like EOG, ECG, EMG, and power lines, as illustrated in Table 4. This spatial limitation—around 1 cm—becomes more pronounced with increased electrode-brain distance, resulting in signal attenuation and potential performance drawbacks for the system. Moreover, EEG signals’ non-stationary nature means their frequency and spectral content shift over time, which can further complicate signal analysis. Despite these challenges, the advantages of EEG, such as its ease of use and the safely harvested non-invasive data, are invaluable, especially in biomedical applications (Arpaia et al., 2023; Liu et al., 2024). Two feature extraction methods in the time domain, LP and ICA, stand out, allowing for the derivation of valuable insights like event-related potentials, mean, standard deviation, and fractal dimension (FD).

**TABLE 4:** Different techniques to analyze EEG signals. Reprinted with permission from (Luján et al., 2021).

Domain of analysis	Feature extraction method	Feature
Time	• Linear prediction (LP)	• Event-related potentials (ERP)
	• Independent component analysis (ICA)	• Statistics of signal power (mean, standard deviation, 1st difference, 2nd difference, entropy, ANOVAS)
		• Hjorth features (activity, mobility, complexity)
		• Fractal dimension (FD)
		• High order crossings (HOC)
Frequency	• Fast Fourier transform (FFT)	• Band power
	• Short-time Fourier transform (STFT)	• High order spectra (HOS)
	• Spectrogram	
	• Autoregressive method (ARM)	
	• Eigenvector	
Time-Frequency	• Wigner Ville distribution	• Combination of time and frequency features
	• Scalogram	
	• Hilbert-Huang spectrum	
	• Discrete wavelet transform (DWT)	
	• Wavelet packet decomposition (WPD)	
Spatial-Time-Frequency	• In multielectrode analysis, the spatial dimension is calculated by the geometrical position of the electrodes	• Combination of time and frequency features

## 5 Prosthetic limb fNIRS-Based control

Functional Near-Infrared Spectroscopy employs near-infrared light to probe cerebral activity, capitalizing on the transparency of human tissues in the near-infrared range (Von Lühmann et al., 2021). Contrastingly, while both fNIRS and EEG are non-invasive, they differ significantly in resolution: fNIRS provides high spatial resolution (in millimeters) but lags in temporal resolution (on the scale of seconds) opposite to EEG’s rapid temporal sensitivity. Currently, achieving concurrent high temporal and spatial resolutions remains elusive for clinical applications. The fNIRS mechanism depends on the differential absorption and scattering characteristics of NIR light in brain tissue (Chen et al., 2020; Pinti et al., 2020). In this technique, NIR light is exposed to the scalp, passing through to interact with cranial tissues. The resulting diffuse reflectance is then captured by detectors, exploiting the minimal absorption of NIR light by water and lipids to achieve tissue penetration (Althobaiti and Al-Naib, 2020; Althobaiti et al., 2018; McGhie and Aldrich-Wright, 2022).

### 5.1 fNIRS-based control architecture

**FIGURE 7:**
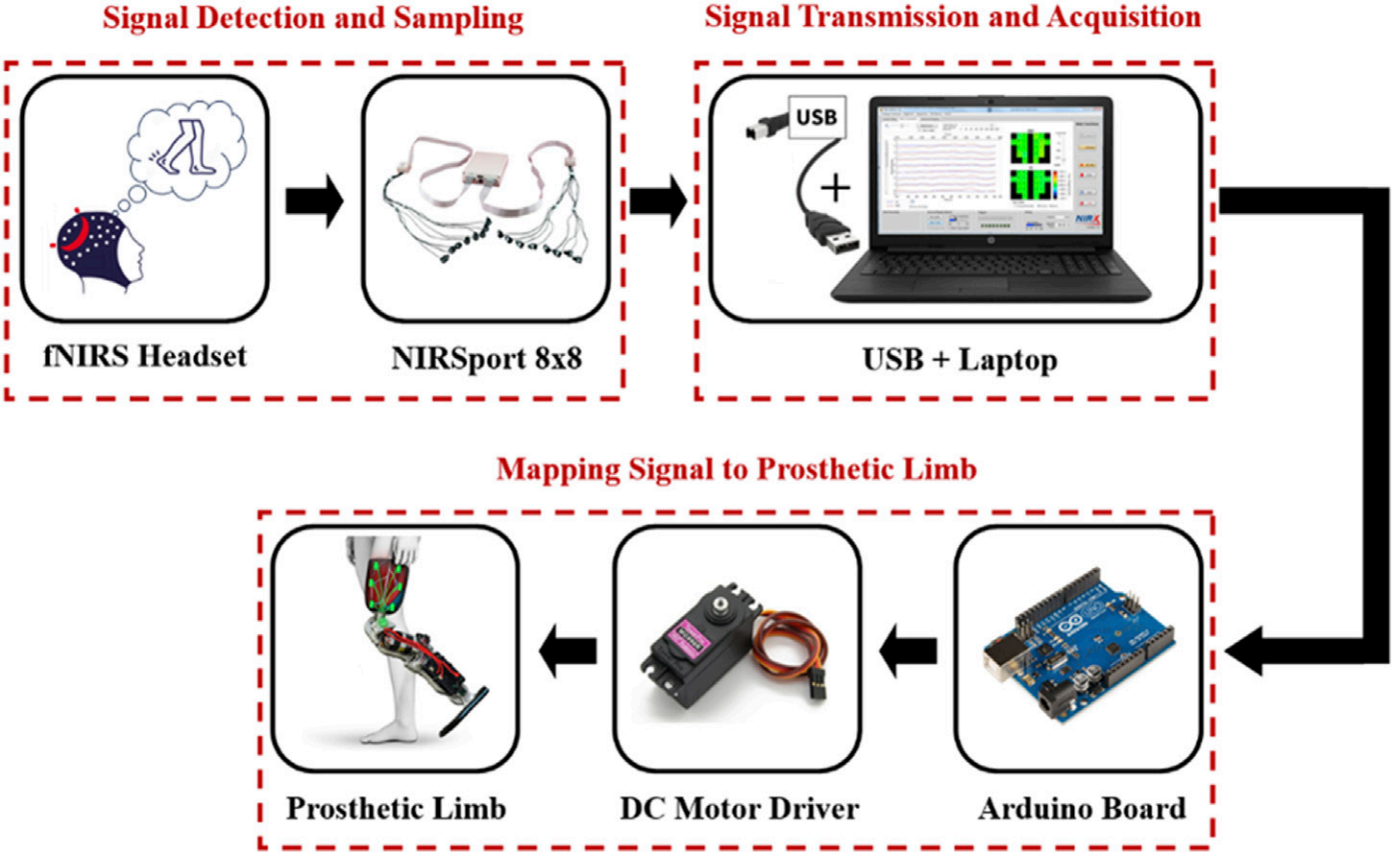
Example of the workflow of fNIRS-based control architecture.

The control architecture utilizing fNIRS technology for prosthetic legs is depicted in Figure 7. The architecture encompasses a series of sequential phases including signal acquisition, processing, feature extraction, and classification. The subsequent sections will delineate the fundamental principles and methodologies employed in each of these phases.

#### 5.1.1 Signal Detection and Sampling in fNIRS systems

fNIRS systems consist of LEDs, detectors, and processing circuits. LEDs emit light onto localized tissue areas at various wavelengths, and detectors capture the back-scattered light, with interactions varying within the near-infrared (NIR) spectrum. Optodes, combining sources and detectors, facilitate these interactions. LEDs are favored for their low power consumption, reliability, and long lifespan, typically using wavelengths of 660, 670, 700, 850, 870, and 940 nm. Laser diodes, such as GaAs/AlGaAs and VCSELs, are possible alternatives to LEDs, offering quite low energy consumption, high-intensity coherent output, and high peak power, commonly used in the 850 nm range and from 750 to 980 nm. As light traverses through human tissue, its intensity diminishes significantly, arriving at detectors ranging from sub-milliwatts to picowatts, requiring highly sensitive detectors to accurately capture these attenuated signals. Notable examples include charge-coupled device cameras, photomultiplier tubes, avalanche photodiodes, and light-sensitive diodes. Particularly in fNIRS systems, silicon photomultipliers, known for their high gain, rapid acquisition speed, exceptional responsivity, and very high sensitivity are being utilized, as detailed in Refs. (Almajidy et al., 2020; Von Lühmann et al., 2021; Nareshkumar, 2022).

Although commercial fNIRS systems are advanced, they lack FDA approval, and standard protocols for device calibration, signal processing, data analysis, and statistical methods are not established (Yücel et al., 2021). The main challenge is that placing multiple sources and detectors on the scalp can be time-intensive, especially with hair, and full head coverage is impractical due to the large number of optodes required (Pfeifer et al., 2017; Quaresima and Ferrari, 2019). Enhancements to headgear design and attachment methods are critical. Initiatives like the study by Liu et al. (2022) address limitations such as traditional equipment being expensive, cumbersome, and complex. They introduced a compact silicon photomultiplier (SiPM) detector that is compact, immune to the magnetic field and has high gain, making it suitable for monitoring brain activity. This design facilitates expanding the array of sources and detectors for high-density recordings, with placement based on the target application and the region of interest (ROI), following the 10–20 system for electrode positioning.

Challenges in fNIRS signal interpretation stem from light traversing various brain tissues and extracerebral artifacts from the scalp and skull. Standard fNIRS captures a mix of cerebral and non-cerebral signals, with superficial layers introducing significant noise. Correcting these measurements is complex due to dynamic signal fluctuations related to probe distance and internal and external biological noises. To isolate cerebral signals from noise, researchers use short-channel subtraction. fNIRS systems employ long separation channels (∼3 cm) for assessing cortical hemodynamic changes and short separation channels (∼1.5 cm) for filtering noise. Short channels measure extracerebral signals to reduce noise by subtracting these readings from long channel signals. This process, while not optimally standardized, shows enhanced signal fidelity with minimized source-detector distances of short channels, highlighting their importance in optimizing fNIRS measurement reliability (Brigadoi and Cooper, 2015; Zhou et al., 2020; Noah et al., 2021; Paranawithana et al., 2022).

fNIRS technology has three modalities: time-domain, continuous wave, and frequency-domain. Time-domain offers high spatial resolution and depth but is costly and bulky. Continuous wave is portable and affordable with a high sampling rate but has limited depth and can’t separate absorption from scattering well. Frequency-domain balances accuracy, sampling rate, and depth. Researchers must choose based on their study’s needs, considering factors like resolution and cost, as summarized in this Ref. (Althobaiti and Al-Naib, 2020).

In a study by Almulla et al. (2020), continuous wave fNIRS was used to evaluate brain hemodynamic responses in the motor cortex during standing and sitting tasks. The participants, nine in total, were instructed to perform real and imagined movements associated with these tasks across five trials. Statistical parametric mapping (SPM) analysis confirmed bilateral activation of oxyhemoglobin for both actual movements and imagined tasks. Notably, sitting tasks elicited higher oxyhemoglobin activation than standing tasks, consistent across all measurement channels in the two experiment sets. Furthermore, six features were extracted from the pre-processed HbO signals: signal mean, signal slope, signal skewness, signal kurtosis, signal variance, and signal minimum. Analysis using various classifiers found that the combination of signal slope and signal variance yielded high accuracy for both real and imagined task trials. These results have the potential to advance rehabilitation practices for lower limbs.

In a subsequent study by Almulla et al. (2022), the fNIRS system was employed to investigate brain hemodynamics associated with postural tasks. This study specifically focused on the observation and motor imagination of such tasks. The research involved 13 healthy participants, each performing five trials of standing balance tasks across three experimental conditions: Action Observation (AO), Motor Imagery (MI), and a combination of both (AO+ MI). The findings revealed significant activation in prefrontal and motor regions during dynamic and static standing tasks, particularly noticeable in the combined AO+ MI and MI settings. While the AO condition alone also led to activation, the combined AO+ MI condition elicited higher activation patterns, especially within the frontopolar area during more demanding balance tasks. Additionally, this combination condition demonstrated significant engagement of the premotor and supplementary motor cortices, which play a crucial role in balance control. Compared with the isolated AO and MI conditions, the AO+ MI setup resulted in the most pronounced activation. These findings not only align with previous research but also underscore the effectiveness of fNIRS as a valuable tool within the realm of rehabilitation diagnostics.

#### 5.1.2 Signal Transmission and Acquisition

The process of acquiring signals in fNIRS involves the use of optodes to pick up brainwave activity. As the optodes detect these signals, they’re subsequently amplified and converted into a digital format for transmission. Typically, a USB connection facilitates the transfer of these digitized signals to a computer for monitoring and analysis. Specialized software, such as NIRStar or Aurora, handles the collection of raw data, allowing for real-time visualization during the recording phase. The data is then stored on the computer system for further manipulation. This raw data undergoes an initial phase of pre-processing, where it’s cleaned and filtered to remove any unwanted noise or artifacts that could potentially skew results. For the classification process, advanced analytical tools like MATLAB or Python are utilized. Here, the technique for brainwave analysis adheres to a framework which is depicted in Figure 5. Following this, relevant features are meticulously extracted from the pre-processed signals to accurately characterize them, and these features serve as the input for various classification algorithms. The classification stage is vital, as it distinguishes different brainwave patterns and correlates them to specific instructions for practical applications. Among the numerous classifiers available, some of the most frequently employed include Linear Discriminant Analysis (LDA), Support Vector Machine (SVM), Quadratic Discriminant Analysis, and k-nearest Neighbor. These classifiers have been verified as effective in numerous studies, as indicated by the references (Pfeifer et al., 2017; Khan R. A. et al., 2018; Klein and Kranczioch, 2019).

#### 5.1.3 Mapping signal to prosthetic leg

fNIRS is emerging as a promising method for controlling bionic prosthetic limbs due to its low susceptibility to noise and immunity to electrical interference, advantages over other brain-computer interface technologies. In a typical setup, a microcontroller, such as the Arduino Uno, retrieves the preprocessed brainwave data from the computer. Its primary role is to interpret these brainwave signals, translating them into precise commands that drive the prosthetic leg’s movements. A DC motor, under the command of the microcontroller, articulates the leg, effectively converting cognitive intentions into physical motion.

### 5.2 fNIRS technology: New controlling approaches

Recent studies have explored the use of fNIRS to enhance lower prosthetic limbs. Möller et al. (2019) assessed brain activity in 29 transfemoral amputees and 16 healthy subjects during level walking via fNIRS. The authors posited that amputees would exhibit greater brain activity than the healthy subjects. Subjects with transfemoral amputations were dichotomized into two groups: those with non-microprocessor-controlled prosthetic knees (n = 14) and those with microprocessor-controlled prosthetic knees (n = 15). The findings indicated increased cortical brain activity in amputees with non-microprocessor-controlled knees during ambulation, suggesting indirect benefits of microprocessor-controlled knees in reducing cerebral exertion in amputees (Möller et al., 2019).

Furthermore, Li et al. (2020) investigated a prediction method for walking intentions using fNIRS measurements to refine the control commands of walking assistive devices. The cerebral hemoglobin signal from 30 subjects was captured using fNIRS technology, and subsequently processed to extract the Teager-Kaiser energy operator. The authors employed a gradient boosting decision tree (GBDT) model for real-time detection, achieving high accuracy in discerning walking intention with a false positive rate of 2.91%, a true positive rate of 100%, and a detection latency of 0.39 ± 1.06 s. The study confirmed the practicality of using fNIRS to decode self-paced walking intentions (Li et al., 2020).

**FIGURE 8:**
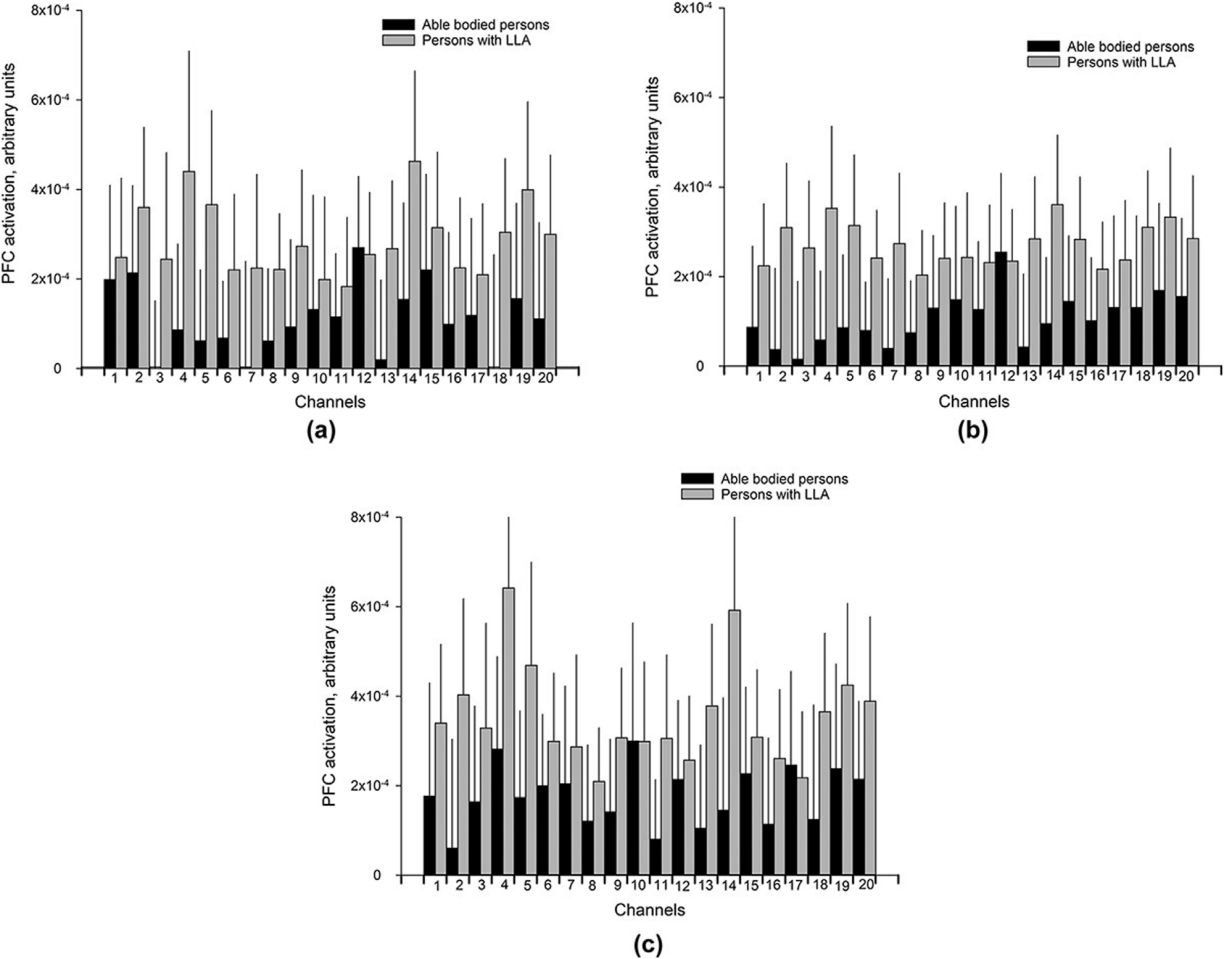
Bar charts of the mean PFC activation (Arbitrary Unit) with 95% confidence intervals (shown as light gray lines) for each of the 20 channels for different walking conditions **(A)** UW (Usual walking with self-selected walking speed); **(B)** WCT (walking and carrying a tray with two cups filled with water), and **(C)** WUT (walking on uneven terrain). Reprinted with permission from (Schack et al., 2022).

Schack et al. (2022) utilized functional near-infrared spectroscopy to assess walking performance and prefrontal cortical (PFC) activity in 33 healthy participants and 39 lower limb amputees. The study investigated three conditions: walking on even terrain, walking while carrying a tray holding two cups of water and walking on uneven terrain. The research compared PFC activity between the two groups—healthy subjects and amputees—concluding that PFC activity increased for amputees during ambulation on both even and uneven surfaces as depicted in Figure 8. This figure clarifies that, across all conditions, each graph segment (a, b, c) juxtaposes the PFC metrics of healthy subjects against those of amputees. This indicates that lower limb amputees require heightened cognitive attention during walking, as their PFC activity levels exceed those of healthy individuals Schack et al. (2022).

### 5.3 Advantages and disadvantages of fNIRS for controlling prosthetic

fNIRS offers the significant advantage of collecting data on prosthetic use with high spatial resolution, as illustrated in Figure 6. Its limitation, however, lies in its low temporal resolution. Despite this drawback, the characteristics of fNIRS—portability, non-invasiveness, and cost-effectiveness—make it a viable option for a range of applications. One notable limitation of fNIRS concerns its penetration depth; it does not extend deeply into the inner cortical areas. Additionally, signal acquisition is susceptible to artifacts, such as those caused by movement. There can also be propagation delays when capturing hemodynamic responses linked to specific neuronal activities (Xu et al., 2021).

In comparing functional near-infrared spectroscopy to other neuroimaging techniques such as EEG, MRI, and PET, fNIRS stands out for its balance of cost, accessibility, and user-friendliness. Unlike MRI and PET, fNIRS is non-invasive, portable, and significantly less expensive, making it a more accessible option for continuous use in controlling prosthetic limbs. Its temporal resolution surpasses that of MRI and PET, though it falls short of EEG, and while its spatial resolution is moderate, it remains sufficient for identifying cortical activation patterns pertinent to prosthetic control. Ethical considerations, including informed consent, neural data privacy, and potential psychological impacts of long-term BCI integration, are of paramount importance. Ensuring patient safety through rigorous testing to rule out adverse effects contributes to the responsible deployment of fNIRS in clinical and non-clinical settings.

Unlike EEG, fNIRS is less affected by scalp-sourced electrical noise and offers better spatial resolution, though it has slower temporal resolution and is limited by light penetration, affecting the depth of measurable neural activity. These characteristics position fNIRS as a promising, non-invasive alternative for real-time applications, combining practicality with a balance of spatial and temporal data quality.

## 6 Combining EEG and fNIRS to control lower limb prosthetics

The potential of EEG and fNIRS to uncover brain activity has captured the interest of scientists in recent years. As a result, numerous studies have evaluated the feasibility of employing EEG and fNIRS for control purposes in gait rehabilitation and brain-computer interface applications (Al-Quraishi et al., 2018; Tariq et al., 2018). Researchers have begun to employ a hybrid system combining EEG and fNIRS as a non-invasive hybrid technique, introducing innovative approaches in BCI technology (Khan et al., 2021). The primary benefit of this fusion is the ability to compensate for the individual limitations of each technique through their integration.

**FIGURE 9:**
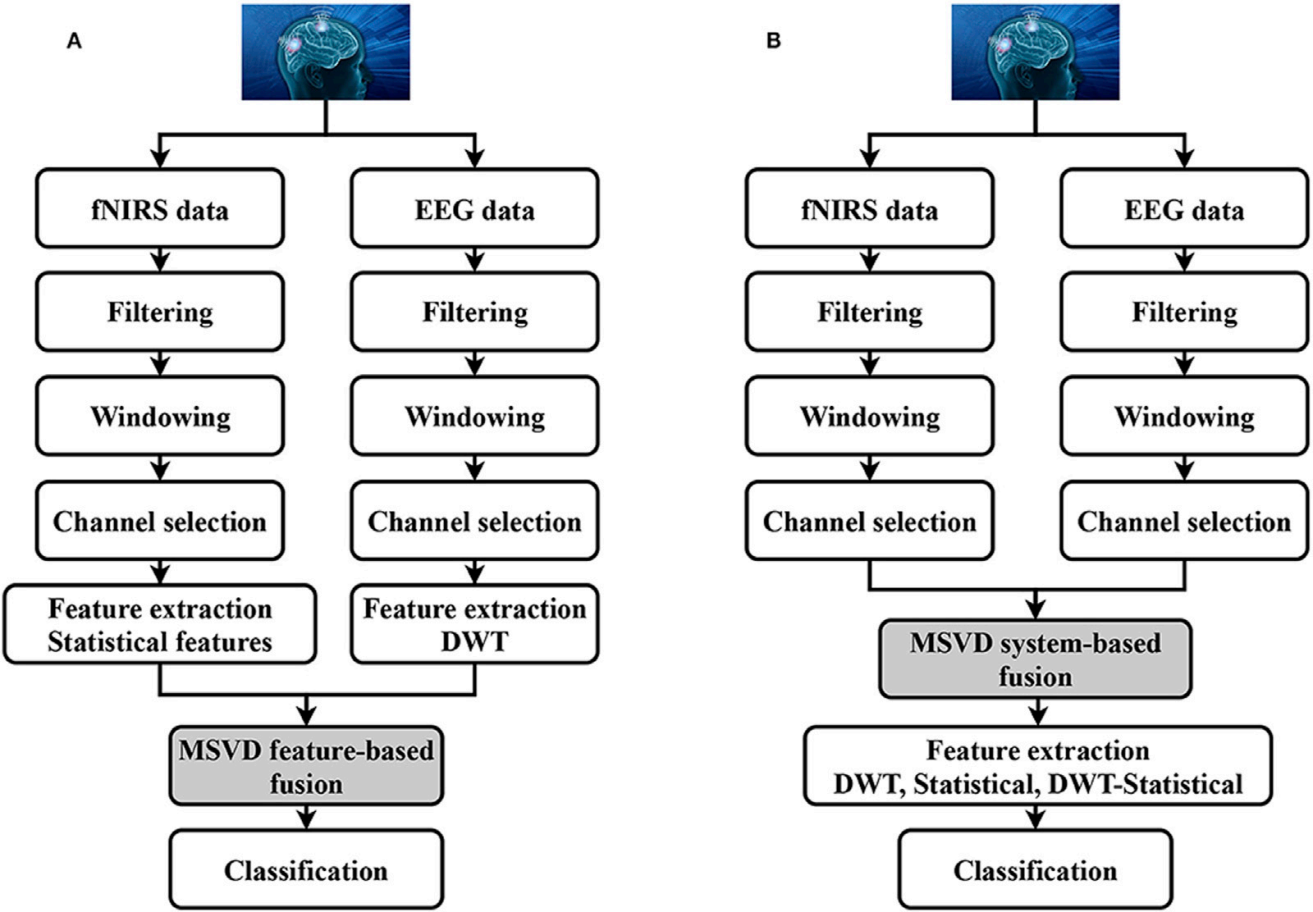
Block diagram of hybrid EEG-fNIRS system. Reprinted with permission from (Khan and Hasan, 2020).

For example, Khan and Hong (2017) proposed a hybrid EEG-fNIRS system capable of interpreting eight, which is quite a significant number, of distinct brain commands for BCI applications, as depicted in Figure 9. Their methodology attained a decoding accuracy of 75.6% for four commands using fNIRS and an 86% accuracy rate for another four commands derived via EEG. It is worth noting that such a system is quite complex and integrating and processing data from two different modalities is also complex and requires advanced algorithms. Moreover, effective use of the system requires significant user training to achieve optimal performance. Furthermore, the research also identified certain constraints, such as the necessity for manual feature extraction, which could influence classification precision.

Multiple studies have assessed the efficacy of integrated EEG-fNIRS systems in comparison to the individual use of each technology (Ahn and June 2017; Cicalese et al., 2020). For example, Li et al. (2017) reported that the combined EEG-fNIRS approach yielded a marked increase in classification accuracy, reaching around 90%, superior to that of either EEG or fNIRS when used separately. Nonetheless, the integration presents salient challenges: foremost are the delayed hemodynamic response inherent to fNIRS, which compromises temporal resolution, and the intricate setup required for colocating fNIRS optodes with EEG electrodes (Li et al., 2017). In seeking to overcome such limitations, Qiu et al. (2022) introduced a novel multimodal fusion framework that harnesses both EEG and fNIRS. This framework utilizes advanced feature extraction and selection across multiple domains to enhance BCI functionality and ensure greater classification precision. Their methodology underwent empirical testing through motor imagery (MI) and mental arithmetic (MA) tasks within an EEG-fNIRS framework. The results underscore the superiority of this fusion technique, demonstrating classification accuracies of 96.74% in the MI task and an exceptional 98.42% in the MA task (Qiu et al., 2022).

**FIGURE 10:**
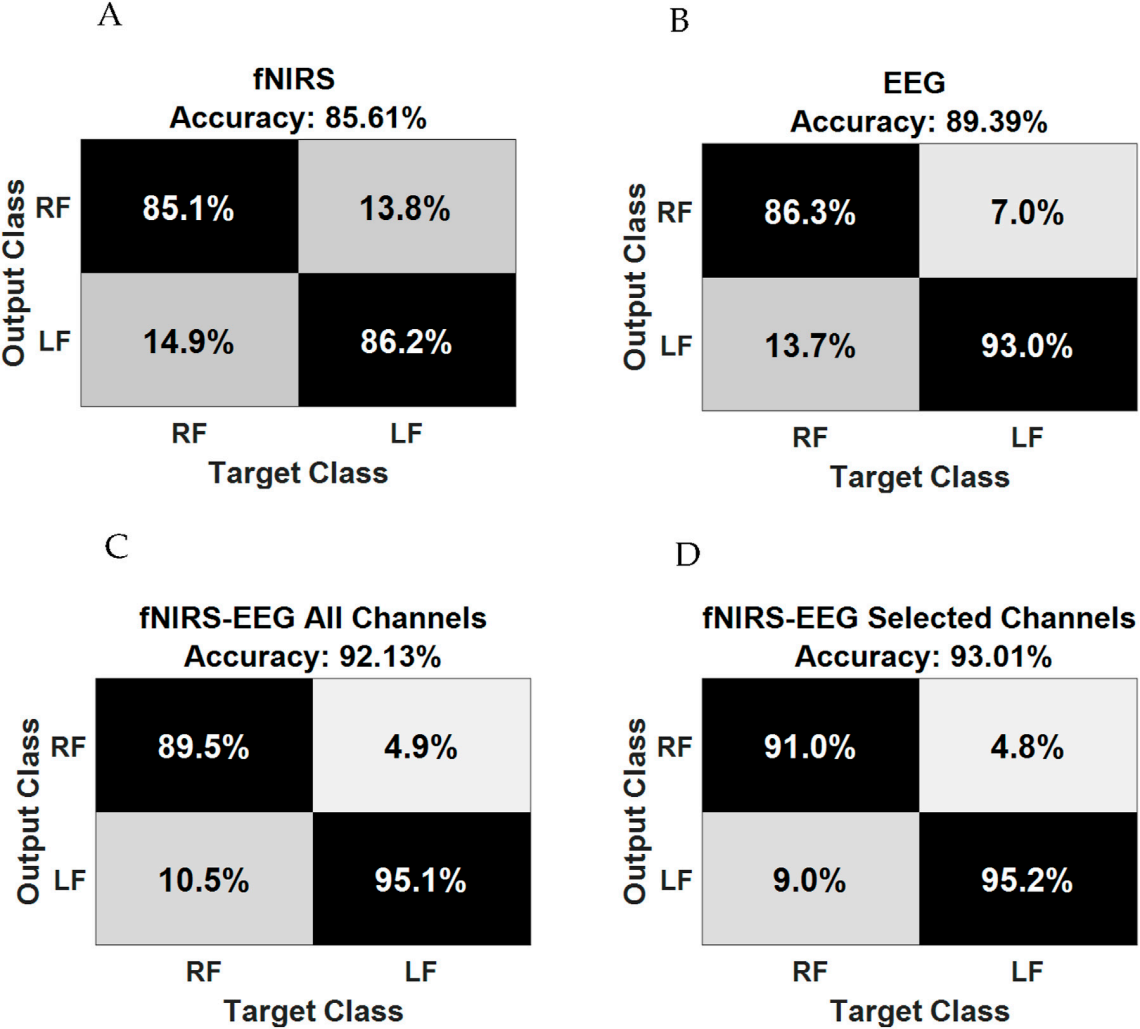
Confusion matrix of **(A)** fNIRS alone; **(B)** EEG alone; **(C)** fNIRS–EEG based on all channels, and **(D)** fNIRS–EEG based on selected channels. Reprinted with permission from (Al-Quraishi et al., 2021).

In a notable study, Al-Quraishi et al. (2021) explored the interplay between brain activity and hemodynamic responses using the multimodal neuroimaging capabilities of EEG and fNIRS. The research involved experiments that focused on ankle joint movements, with twenty participants serving as subjects. These individuals were examined using a configuration of twenty EEG electrodes and thirty-two fNIRS optodes, which were strategically positioned over the motor cortex. The EEG signals revealed an event-related desynchronization (ERD) in the 8–11 Hz frequency range. Concurrently, the fNIRS data were analyzed to measure variations in oxygenated hemoglobin (oxyHb) concentration. Pearson’s correlation coefficient was utilized to evaluate the relationship between oxyHb changes and the ERD during ankle joint movements. The analyses produced compelling evidence of a negative correlation between the ERD and hemodynamic responses. Notably, channels Ch28, Ch25, Ch32, and Ch35 from fNIRS exhibited significant correlations with EEG channels Cz and Cpz, particularly during right ankle joint movements. These channels were subsequently utilized for classifying movements with enhanced precision. Statistical analyses solidified the understanding that the dual-modality approach surpassed the performance of the singular modalities in classifying ankle joint movements, achieving an average accuracy of 93.01% ± 5.60% with a *p*-value <0.01. These results are presented in Figure 10 (Al-Quraishi et al., 2021), which includes a confusion matrix for both individual and hybrid modalities.

While the synergistic EEG-fNIRS system demonstrates superior performance in comparison to each standalone system, there remains a significant pursuit for advancing the system’s accuracy through the development of innovative processing techniques and classification methodologies. Table 5 presents a comparison of selected studies employing hybrid systems within their experimental frameworks. The combination of EEG and fNIRS modalities for prosthetic applications presents a promising avenue to improve brain-computer interfaces, yet it brings forth several challenges, especially in configuring both fNIRS optodes and EEG electrodes. A notable constraint arises from the rather complex setup and arrangements of these modalities, leading to difficulties in managing backend wiring. The integration of numerous electrodes and optodes for a large number of channels requires meticulous handling to prevent wire entanglement, ensuring a dependable connection without compromising the user’s mobility or comfort. Wireless connections could be used to avoid using wires and optical fibers, but at the expense of the signal quality and the added complexity of the overall system. Another significant drawback is the considerable footprint of the combined EEG and fNIRS system. The necessity for multiple electrodes and optodes across the scalp can result in a cumbersome and bulky configuration, potentially impeding the widespread adoption of prosthetic applications. Streamlining the design to reduce overall size and weight while preserving functionality becomes a vital consideration to enhance user acceptance and practicality. It is worth mentioning that mitigating the risks of signal contamination from external light sources or electromagnetic interference with the sources and detectors of the hybrid system is essential for obtaining reliable data in real-world scenarios.

**TABLE 5:** Summary advantages and disadvantages of selected papers that use hybrid systems.

Author, year	Advantages	Disadvantages
Von Lühmann et al. (2017)	• Modular design	• Limited channel numbers, only 13 fNIRS channels, and 8 EEG channels
	• Both wet and dry EEG electrodes can be used	• Relatively limited scalability can only be theoretically scaled up to four modules
	• 3-axis accelerometer for local movement monitoring	• Capability of imaging not demonstrated
	• Shared ADC architecture	• Modules possess a large footprint
		• Not capable of whole scalp sampling
		• The weight of each module is unclear
Kassab et al. (2018)	• 128 fNIRS channels, 32 EEG channels	• Increased size, weight, and cabling as compared to the above system
	• Same advantages as of Ref. (Von Lühmann et al., 2017)	• Same limitations as of Ref. (Von Lühmann et al., 2017)
Lee et al. (2019b)	• 16 EEG channels	• Limited fNIRS channel numbers, only 8 fNIRS channels
	• Custom-designed dry EEG electrodes	• Extensive analog cabling for EEG and fNIRS into the control module
	• Battery operated	• Fixed SDS of 27 mm (sparse spatial sampling)
		• Separate ADCs
		• The total weight of the system is unclear
Hasan et al. (2020)	• High motor classification accuracy of 72.42% ± 3% through fNIRS using the Tree classifier	• Low motor classification accuracy of 52.49% ± 4% through EEG only
		• A hybrid system is highly affected by the low spatial resolution of the EEG system
Arif et al. (2022)	• A novel methodology has been proposed for enhancing the average classification of EEG-fNIRS BCI systems	• A comparison of accuracy based on gender has not been conducted, potentially impacting the overall accuracy
	• The novel classifier with a high average classification accuracy of 91.35%	• A false positive detection can result in some false detection of activity
		• The presence of artifacts affects the performance of the system
Mughal et al. (2022)	• High average accuracy levels of 78.44% for fNIRS, 86.24% for EEG, and 88.41% for hybrid EEG-fNIRS BCI when using the proposed model	• The proposed methodology is computationally costly
		• The proposed algorithm has not yet been implemented for real-time BCI.
Xu et al. (2023)	• The relative activation of the left and right brain regions was significant during the exercise imagination period of the subjects	• Data were collected from healthy subjects

Consideration for user comfort and practicality is also essential in applying non-invasive EEG and fNIRS technologies outside clinical settings. Innovations in sensor design strive for minimal obtrusiveness and optimized wearability, balancing technical efficacy with user-centric factors. Longitudinal studies provide data on ergonomic integration and the clinical relevance of these systems, ensuring they meet healthcare’s patient-centered goals. As the potential of EEG and fNIRS grows, their everyday applicability hinges on addressing these user experience challenges (Das et al., 2016; Park et al., 2020).

The adoption of EEG and fNIRS technologies in clinical settings faces specific challenges that need to be addressed for their optimal utilization. One critical consideration is the choice of EEG device type, which depends on features and applications. For instance, the wireless EEG setup proves beneficial when monitoring patients who need freedom of movement, as opposed to clinical EEG devices where patients are required to remain stationary during recording to prevent signal distortion. Overcoming the challenge of signal distortion during movement hinges on the design mechanism employed. In a wireless EEG setup, the system is specifically designed to measure brain activity during motion with minimal signal loss, ensuring accurate recordings even when patients are mobile. Despite the ambition for a system with high temporal and spatial resolutions in clinical settings, the realization of such a goal remains a challenge. Striking a balance between achieving superior resolution and maintaining practicality in clinical applications poses a considerable obstacle. Researchers and developers continue to explore ways to enhance the resolution of EEG systems for clinical use, but the current technological landscape presents limitations in achieving the desired level of both temporal and spatial precision. In the case of fNIRS technology, its utilization in clinical settings relies on exploiting the absorption and scattering properties of near-infrared (NIR) light to gather information about brain activity. While fNIRS provides valuable insights, challenges persist in optimizing its accuracy and reliability for clinical applications. Overcoming these obstacles involves refining the technology’s sensitivity and specificity, ensuring robust performance across different patient populations and clinical conditions. Clinical trials with large groups of subjects with various clinical situations are needed. One of the important limitations of such systems can be foreseen when they are integrated with the real prosthesis due to the socket designs that offer comfort to the patients. Hence, the designers of prosthetics and hybrid (fNIRS, EEG, and EMG) system designers should consider this very important challenge. For instance, miniaturized EMG sensors have been indeed integrated into the sockets offering an excellent starting point for such research problems (Su et al., 2023).

The recent advances in machine learning algorithms used in signal processing have a considerable impact in enhancing the performance of hybrid systems and getting higher classification accuracy. The common machine learning (ML) algorithms used to augment classification accuracy include linear discriminant analysis (LDA), support vector machine (SVM), artificial neural networks (ANNs), etc. A study by FJ Ramírez-Arias investigated the effect of ML algorithms in EEG signals classification (Ramírez-Arias et al., 2022). They aimed to relate the motor movements, including right and left hands, fists, feet, and relaxation, to their signals. Therefore, EEG datasets of 30 Physionet subjects were utilized to train and evaluate nine ML algorithms. The results showed how the advances in ML algorithms yielded higher classification accuracies. For instance, LDA, SVM, and ANNs models reported accuracies of 92.3%, 98%, and 99.9%, respectively (Ramírez-Arias et al., 2022).

Similarly, ML algorithms exhibit remarkable performance and superior accuracy in the classification of hybrid EEG-fNIRS systems. When information obtained from fNIRS and EEG were used in ML algorithms, the hybrid EEG-fNIRS BCI system’s efficacy and accuracy were notably improved, as proved by Padmavathy et al. They showed that the novel Deep Neural Network yielded the best accuracy rate of 84% for integrated EEG-fNIRS compared to independent EEG and fNIRS with an accuracy of 74% and 75%, respectively. Although multi-modal recording techniques can greatly improve system performance, tremendous advances in machine learning algorithms and BCIs have greatly improved the performance of these techniques (Padmavathy et al., 2020).

Though a considerable amount of research has been carried out in the last decade, there are several possible directions for future research. (i) Understanding the neural control mechanisms by investigating neural signals for lower limb movements using EEG and fNIRS. The approach for that could be by identifying specific EEG and fNIRS signal patterns for various movements (e.g., walking, running, climbing stairs) and developing algorithms to decode these signals into commands for prosthetic control. More importantly, training users to generate consistent neural signals through motor imagery tasks. (ii) Real-time signal processing and feedback with the objective of ensuring real-time processing of EEG and fNIRS signals for immediate prosthetic control by developing real-time signal processing pipelines for filtering, preprocessing, and decoding signals. (iii) Integration with existing prosthetic technologies by collaborating with prosthetic manufacturers to integrate neural control modules. Moreover, conduct user trials to validate the integrated system’s performance. (iv) Clinical trials and validation by design and conduct clinical trials with amputees using the hybrid BCI prosthetic system. Furthermore, measuring the outcomes like control accuracy, user comfort, ease of use, and functionality. (v) Rehabilitation and training programs by designing training protocols for users to generate consistent neural signals and provide rehabilitation sessions to enhance motor imagery capabilities. Finally, (vi) the ethical and regulatory considerations should be looked at to ensure compliance with medical device regulations and safety standards. Moreover, a special emphasis should be on data security and user consent concerns.

Furthermore, the cost implications and accessibility issues associated with integrating EEG and fNIRS technologies into prosthetic limbs are essential aspects to consider. The initial costs of acquiring and setting up these neurotechnologies, coupled with ongoing operational and training expenses, pose significant financial challenges. To enhance accessibility, potential strategies include seeking government funding and subsidies, advocating for insurance coverage, improving manufacturing processes to scale production and reduce costs, and fostering public-private partnerships to support research and development. By implementing these measures, we can make these advanced prosthetic technologies more affordable and accessible to a broader population, ultimately improving the quality of life for individuals requiring prosthetic limbs.

In the future, it will be important to focus on the reliability and upkeep of EEG and fNIRS-based prosthetics, ensuring signal stability over time, equipment durability, and the necessity for recalibration or updates. This attention could prove beneficial for both researchers and clinicians working with these technologies.

In addition to EEG-fNIRS hybrid BCIs, other hybrid systems such as EEG-EMG and fNIRS-EMG have shown promise in the field of lower limb prosthetics. These systems leverage the strengths of both modalities to enhance control mechanisms (Brambilla et al., 2021; Hong and Khan, 2017). However, our review prioritizes EEG-fNIRS hybrid BCIs due to their unique ability to provide comprehensive neural and hemodynamic insights, which are pivotal for the development of advanced prosthetic controls. Future research should also explore the other hybrid systems to fully understand their potential.

## 7 Conclusion

In conclusion, lower limb amputees contend with a significant impairment that adversely impacts their quality of life, prompting extensive research into enhancing lower prosthetic limbs. The escalating global cases of lower limb amputations underscore the urgency for innovative solutions. Recent advancements have explored combining different brain signal measurement systems, with a particular focus on fusing fNIRS and EEG to create a hybrid BCI. This approach holds tremendous potential, evidenced by a high motor classification accuracy of 72.42% ± 3% using the Tree classifier. Moreover, the hybrid system not only significantly reduces computational burden but also achieves classification accuracy with high reliability, comparable to existing literature. Novel methodologies have been proposed for EEG-fNIRS BCI systems, leading to a remarkably high average classification accuracy of 91.35%. While the review encapsulates recent progress in combining fNIRS and EEG modalities for natural, sensitive, and responsive control of prosthetic devices, the identified advancements emphasize the transformative impact of these technologies. This opens avenues for breakthroughs in the rehabilitation field and underscores the promising trajectory for further research, potentially revolutionizing prosthetic control for enhanced patient outcomes.
